# L-dopa response pattern in a rat model of mild striatonigral degeneration

**DOI:** 10.1371/journal.pone.0218130

**Published:** 2019-06-10

**Authors:** Christine Kaindlstorfer, Nadia Stefanova, Joanna Garcia, Florian Krismer, Máté Döbrössy, Georg Göbel, Kurt Jellinger, Roberta Granata, Gregor Karl Wenning

**Affiliations:** 1 Division of Neurobiology, Department of Neurology, Medical University Innsbruck, Innsbruck, Austria; 2 University Medical Centre Freiburg, Department of Neurosurgery, Freiburg, Germany; 3 Medical University Innsbruck, Department of Medical Statistics, Informatics and Health Economics, Innsbruck, Austria; 4 Institute of Clinical Neurobiology, Vienna, Austria; Florey Institute of Neuroscience and Mental Health, The University of Melbourne, AUSTRALIA

## Abstract

**Background:**

Unresponsiveness to dopaminergic therapies is a key feature in the diagnosis of multiple system atrophy (MSA) and a major unmet need in the treatment of MSA patients caused by combined striatonigral degeneration (SND). Transgenic, alpha-synuclein animal models do not recapitulate this lack of levodopa responsiveness. In order to preclinically study interventions including striatal cell grafts, models that feature SND are required. Most of the previous studies focused on extensive nigral and striatal lesions corresponding to advanced MSA-P/SND. The aim of the current study was to replicate mild stage MSA-P/SND with L-dopa failure.

**Methods and results:**

Two different striatal quinolinic acid (QA) lesions following a striatal 6-OHDA lesion replicating mild and severe MSA-P/SND, respectively, were investigated and compared to 6-OHDA lesioned animals. After the initial 6-OHDA lesion there was a significant improvement of motor performance after dopaminergic stimulation in the cylinder and stepping test (p<0.001). Response to L-dopa treatment declined in both MSA-P/SND groups reflecting striatal damage of lateral motor areas in contrast to the 6-OHDA only lesioned animals (p<0.01). The remaining striatal volume correlated strongly with contralateral apomorphine induced rotation behaviour and contralateral paw use during L-dopa treatment in cylinder and stepping test (p<0.001).

**Conclusion:**

Our novel L-dopa response data suggest that L-dopa failure can be induced by restricted lateral striatal lesions combined with dopaminergic denervation. We propose that this sequential striatal double-lesion model replicates a mild stage of MSA-P/SND and is suitable to address neuro-regenerative therapies aimed at restoring dopaminergic responsiveness.

## Introduction

Neurotoxic lesion models are valuable to study motor symptoms and investigate neuroanatomical correlations and therapeutic approaches in neurodegenerative diseases including multiple system atrophy [1]. Multiple system atrophy (MSA) is an adult onset, rapidly progressive neurodegenerative disease characterized by progressive autonomic failure in combination with parkinsonism and/or cerebellar symptoms and pyramidal features [2]. Disease onset is in the sixth decade, both sexes are equally affected, and mean survival is estimated to be 6–9 years [1,3–6]. According to the second consensus criteria in 2008, MSA patients can be categorized into the Parkinson (MSA-P) or cerebellar (MSA-C) clinical variants depending on the predominant motor presentation of either parkinsonism or cerebellar features [7]. Both variants of MSA are neuropathologically characterized by neuronal loss in multiple brain regions including the autonomic, striatonigral and olivopontocerebellar systems. The degeneration of dopaminergic neurons in the substantia nigra and the medium sized spiny projection neurons of the striatum is predominant in MSA-P and is referred to as striatonigral degeneration (SND), while neurodegeneration in MSA-C is most prominently observed in the olivopontocerebellar system resulting in olivopontocerebellar atrophy (OPCA) [8–10]. However, both systems are usually affected within one individual. Histologically, both motor variants of MSA feature a common and disease-specific cellular pathology of α-synuclein (α-SYN) immunoreactive aggregates in oligodendrocytes, which are referred to as (oligodendro-) glial cytoplasmic inclusion bodies [11].

Currently, most translational MSA studies are based on transgenic mouse models overexpressing α-SYN in oligodendrocytes aiming to identify disease modifying agents [12–16]. However, thus far, no established disease modifying treatment is available for MSA and treatment options are generally limited and confined to symptomatic approaches targeting motor symptoms, autonomic dysfunction and occupational therapy to support patients´ independency in activities of daily living [5]. Contrary to PD, parkinsonism in MSA-P is typically resistant or poorly responsive to dopamine replacement therapy and if MSA-P patients respond to L-dopa treatment, the effect is usually transient with a mean L-dopa responsiveness of 3.5 years [1,5,6,17]. L-dopa failure in MSA-P reflects the loss of dopamine receptor bearing medium spiny GABAergic neurons of the striatum as part of SND [17]. Novel experimental models replicating different degrees of basal ganglia degeneration are required to study the relevance of selective basal ganglia atrophy to dopaminergic responsiveness. Although, the existing transgenic mouse models, especially the (PLP)-alpha-synuclein model, exhibit neuronal degeneration in the substantia nigra that gradually affects the striatum over 12 months of age along with motor deficits, the effectiveness and response patterns of L-dopa treatment are difficult to assess using this model [12,18–20]. In contrast, the classical double-lesion rat model of MSA-P/SND exploits two well established neurotoxins, 6-hydroxydopamine (6-OHDA) and quinolinic acid (QA), replicating SND. and is suitable to test dopaminergic responsiveness [21–25].

6-OHDA is a dopamine derivate that enters catecholaminergic terminals via dopamine and noradrenaline reuptake transporters and leads to the production of reactive oxygen species resulting in dopaminergic cell death in the substantia nigra [26]. Degeneration of medium spiny neurons of the striatum can be induced by striatal injection of QA, which is an NMDA receptor agonist resulting in excitotoxicity and cell death [27] complementing the 6-OHDA induced nigral degeneration to replicate SND. Hence, the combination of 6-OHDA and QA causes MSA-P/SND-like lesion pattern of variable extent depending on the site and distribution of injection and the dosage of neurotoxins [28,29]. In the past, MSA-P/SND models have been based on complete dopaminergic denervation of host striatum using medial forebrain bundle (MFB) lesions and—often extensive—striatal damage replicating advanced SND. Despite replication of L-dopa failure in the advanced SND model, the extensive neuronal loss potentially limits the translation to the clinic and the success of transplantation approaches [21–25]. Therefore, a partial double lesion model with stable L-dopa treatment failure and a histopathological situation potentially suitable for neuro-restorative interventions is required. The feasibility of an unilateral partial, sequential striatal double lesion rat model combining a subtotal 6-hydroxidopamine (6-OHDA) lesion followed by a partial striatal quinolinic acid (QA) lesion mimicking early stage MSA-P pathology has been described previously [30]. The aim and achievement of the current study was to further develop and characterize this model using a series of behavioural L-dopa response paradigms and a severe striatal double-lesion control group.

## Materials and methods

### Animals

A total of 54 adult male Wistar rats (Charles River; Sulzfeld, Germany) aged 12 weeks and weighing 200–225 grams at the beginning of the experiment were used in this study. The animals were housed in a temperature- and humidity-controlled environment under a 12-hour light/dark cycle in the animal facility of the Medical University of Innsbruck with water and food *ad libitum*. All efforts were made to minimize the number of animals used and their suffering. This study was approved by the Institutional Animal Care and Use Committee at the Medical University Innsbruck and the Federal Ministry for Science and Research of Austria. All experiments were executed in accordance with the Austrian law.

### Experimental design

All animals underwent the initial striatal 6-OHDA injection. Subsequently, the first behavioural assessment including amphetamine induced rotation and evaluation of dopaminergic responsiveness in cylinder and stepping test using saline/L-dopa treatment (S1 = saline and LD1 = L-dopa treatment) was performed (the details are explained in the section *behavioural analyses*). Animals were then randomized into the three experimental groups:

- group 1 received the centrolateral “severe” striatal lesion with 180 nmol QA- group 1 (n = 19)
- group 2 received the lateral “mild” striatal QA lesion with 120 nmol QA—group 2 (n = 18)
- group 3 did not receive a QA lesion and served as PD/6-OHDA only control group (n = 17)

**Fig 1:**
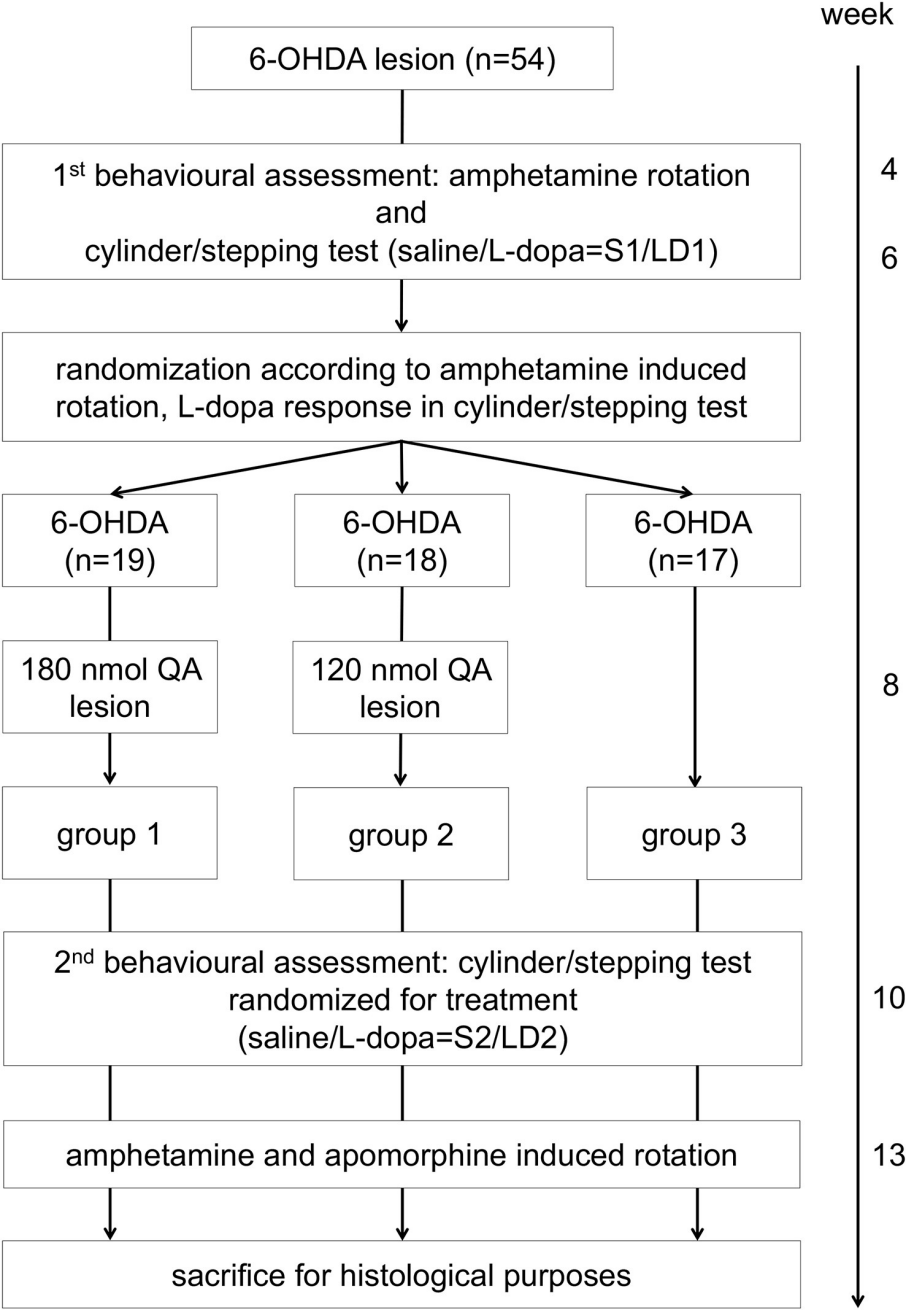
Experimental design. All 54 male Wistar rats underwent 6-hydroxydopamine (6-OHDA) lesion surgery. The first behavioural assessment included amphetamine induced rotation, cylinder and stepping test with saline (S1) or L-dopa (LD1) treatment. Animals were then randomized into the three experimental groups according to rotation behaviour and L-dopa response. Groups 1 and 2 received the additional quinolinic acid (QA) lesions: group 1: 6-OHDA+severe QA and group 2: 6-OHDA+mild QA, while group 3 served as the Parkinson´s disease control group with the 6-OHDA lesion only. Afterwards, the second behavioural assessment including cylinder and stepping test with saline (S2) or L-dopa treatment (LD2), amphetamine and apomorphine induced rotation was performed. Finally, animals were sacrificed for histological purposes.

The second behavioural assessment (2^nd^ behavioural assessment: S2 = saline and LD2 = L-dopa treatment) included amphetamine and apomorphine induced rotation in addition to cylinder and stepping test with and without L-dopa (S2 = saline and LD2 = L-dopa treatment). Finally, animals were sacrificed for histological purposes. The experimental design is shown in Fig 1.

### Stereotactic lesion surgery

Stereotactic lesions were performed targeting the right lateral striatum, which is considered to regulate forepaw, head and orofacial motor behaviour in rodents [31–33]. The concentration of the neurotoxins 6-OHDA and QA (Sigma Aldrich, Steinheim, Germany) were chosen based on previous reports [30] and applied sequentially, with 4 weeks in-between the stereotactic operations. Anteroposterior (AP) and mediolateral (ML) coordinates were set according to bregma and the dorsoventral (DV) coordinate in association to dura [33]. Animals were anaesthetized using isoflurane/oxygen-ventilation (1.5–2.0%, Baxter, USA) and placed in a stereotactic frame (Stoelting, Wood Dale, USA). Fixation was achieved by the tooth- and ear bars of the frame. Animals remained in this position and were anesthetized till the end of surgery. In order to achieve a partial depletion of the nigrostriatal system the previously established terminal partial 6-OHDA lesion protocol was used [30]. Briefly, 3 tracks targeting the right dorsolateral striatum were drilled into the skull and a 10 μl Hamilton syringe (Hamilton Europe, Switzerland) was used to deliver 2 μl of 7μg/μl 6-OHDA (Sigma Aldrich, Steinheim, Germany) at the following coordinates: AP: +1.0/-0.1/-1.2 mm, ML: -3.0/-3.7/-4.5 mm, and DV: -5.0 mm. In all cases, the tooth-bar was at 0.0 mm. The injection rate was 1.0 μl l/min and the syringe was kept *in situ* for 5 min prior retraction. To avoid oxidation of the neurotoxin, 6-OHDA was kept in the dark, on ice and was renewed after each animal. Administration of 0.12M QA (Sigma Aldrich, Steinheim, Germany; dissolved in 0.1M 1x phosphate buffered saline (PBS) solution, pH = 7.4) was delivered into the right striatum by manual infusion using a 26-gauge stainless steel cannula connected to 5μl Hamilton syringe. Animals of group 1 received 180 nmol QA via 3 tracks delivering 3x 0.5μl (2x 0.25μl) of 0.12M QA into the centrolateral striatum at the following coordinates: AP: +1.00/-0.10/-1.20 mm, ML: -3.00/-3.70/-4.50 mm and DV: -5.00 mm. Animals of group 2 received 120 nmol QA via 2 tracks delivering 2x 0.5μl per track into the dorsolateral striatum at the following coordinates: AP: +1.20/-0.40 mm, ML: -3.60/-4.40 mm and DV: -5.50/-4.50 mm for track 1 and DV: -5.80/-6.80 mm for track 2. In all cases the tooth bar was at -2.3 mm. After each deposit of 0.25μl the cannula was kept in place for 4 minutes to allow the neurotoxin to diffuse and reduce the release of QA during retraction of the needle. Animals that showed signs of pain (n = 5) including piloerection, increased back arching, decreased activity, and protecting the painful area received carprofen 4 mg/kg body weight subcutaneously.

### Behavioural analyses

#### Drug induced rotation

Amphetamine- and apomorphine (Sigma-Aldrich, Steinheim, Germany) -induced rotation were used to evaluate the rotation behaviour after 6-OHDA and after QA lesion surgeries. Amphetamine induced rotation was performed 4 weeks after the initial 6-OHDA lesion and at least 3 days prior to L-dopa/saline challenge in cylinder and stepping test at the first behavioural assessment. After QA lesion surgery, amphetamine induced rotation was performed at least 2 days after the L-dopa/saline challenge of the second behavioural assessment. Apomorphine induced rotation was performed at the very end of the behavioural analyses at the second behavioural assessment (3 days after amphetamine induced rotation). After application of either amphetamine or apomorphine, animals were monitored in rotation boxes modelled after Ungerstedt and Arbuthnott [34]. Amphetamine-induced rotation was performed at the first and the second behavioural assessment using 2.5mg/kg amphetamine, delivered by 0.1 ml/100g intraperitoneal injection followed by a 60min assessment period. Apomorphine-induced rotation was performed at the very end of the *intra vitam* analyses in order to avoid receptor desensitization in the lesioned striatum during the experiments. The concentration of apomorphine was 0.75 mg/ml/kg dissolved in 0.2% L-ascorbic acid-saline (Sigma-Aldrich, Steinheim, Germany) with an injection volume of 0.1 ml/100g subcutaneously. Subsequently, animals were monitored for 40 min. Apomorphine solution was stored on ice and protected from light to avoid oxidation. The automated rotation system counted right (ipsilateral) and left (contralateral) body turns separately which was then divided by the minutes of rotation time to calculate the rotations per minute. All animals without rotation behaviour after apomorphine administration, were re-rotated in a second assessment to avoid absent rotations due to failure of injections.

#### Cylinder and stepping test

Lesion and treatment effects of L-dopa were evaluated using cylinder and stepping tests which both assess forelimb akinesia [35,36]. Randomized to treatment (saline or L-dopa) all animals were tested twice at the first and the second behavioural assessment (S1/LD1 = saline/L-dopa treatment at the first behavioural assessment, S2/LD2 = saline/L-dopa at the second behavioural assessment; Fig 1). The concentration of L-dopa (Research Organics, Cleveland, OH, USA) was 6mg/kg plus 10mg/kg benserazide hydrochloride (Sigma-Aldrich, Steinheim, Germany). The volume was 0.1ml/100g and application was performed intraperitoneally 60 min prior to testing. Saline treatment was performed in an analogous mode (0.1ml/100g). After application of either L-dopa or saline each animal performed the cylinder test lasting for a maximum of 4 minutes, followed by the stepping test.

For cylinder test, each animal was placed in a plexiglass cylinder individually (diameter measures 20 cm) and left there to freely explore space. The experiment was performed under conditions of darkness, where red light allowed the recording of animals during the test. Animals were recorded for 4 min and later scored by an experimenter blinded to the identity of the animals. Only animals reaching a total of 20 wall touches and only the first 20 wall touches were considered for analyses. Data of cylinder test are expressed as total wall contacts performed with the left (contralateral to the lesion) and right (ipsilateral to the lesion) forepaw and as percentage of contralateral paw use (wall contacts made with the contralateral paw divided by the sum of wall contacts made with ipsi- and contralateral paw).

For stepping test, an experimenter that was blinded to the lesion and treatment status of the animals performed the test. Animals were held in the hands of the experimenter by fixing the hindlimbs and one of the forelimbs, letting the unrestrained paw reach the surface of the examination table. Then the animals were moved sideways with a mean speed of 0.6m in 5s. Depending on the direction, stepping behaviour was evaluated either in forehand or backhand directions. The experimenter counted the number of adjusting steps in each direction and for each forelimb. The animals were tested 4 times in backhand and forehand direction at each behavioural and treatment assessment to allow calculation of a mean. Data of stepping test are expressed as total adjustment steps and as “limb asymmetry score” (LAS) calculated as follows: ipsilateral steps minus contralateral steps divided by the sum of ipsilateral and contralateral steps. The data ranged from 0–1 with 0 representing no limb asymmetry and 1 representing 100% limb asymmetry.

### Histological examinations

At the end of the second behavioural assessment, animals were sacrificed by an overdose of thiopental via intraperitoneal application (120 mg/kg bodyweight) followed by transcardial perfusion. In order to avoid fixation of blood in the vessels animals were perfused with 50 ml of 0.01M PBS prior to perfusion using 250 ml of 4% paraformaldehyde (PFA) for fixation. After perfusion, brains were quickly removed and post-fixed overnight in 4% PFA at 4°C. Cryoprotection was performed in a solution of 20% sucrose in 0.01M PBS pH 7.4 for at least 24 hours. Subsequently, brains were slowly frozen in 2-methylbutane at -40°C and stored at -80°C until further processing. Six series of sections of 40 μm were cut on a cryostat (Leica, Nussloch, Germany). Immunohistochemical stainings were conducted on free-floating sections according to the “Avidin-Biotin-horseradish-peroxidase-Complex (ABC) method”. On the first day peroxidase blocking was performed using 0.3% H_2_O_2_-PBS solution to reduce endogenous peroxidase activity. Further, sections were pre-incubated in 5% horse serum containing 0.3% Triton X-100 in PBS and an overnight incubation at room temperature with the primary antibody against tyrosine hydroxylase (mouse anti-TH; 1:2500; Sigma-Aldrich, USA) and dopamine- and adenosine 3,5-monophosphate-regulated phosphoprotein (rabbit anti-DARPP-32; 1:20000; generous gift from Prof. Hugh Hemmings, New York). After rinsing sections with PBS, they were incubated with a biotinylated horse anti-mouse/rat secondary antibody (1:200; Vector Laboratories, USA) before they were rinsed again and incubated with avidin-biotin peroxidase solution (ABC Elite; Vector Laboratories, USA) followed by visualization using 3,3’-diaminobenzidin-tetrahydrochlorid (DAB; Merck, Darmstadt, Germany) and 0.01% H_2_O_2_. Finally, sections were mounted on glass slides and covered with entellan under a cover slip.

### Morphological analyses

An observer blinded to the group assignment of the animals performed all stereological investigations using a computer-assisted image analysis system (Nikon E-800 microscope, Nikon digital camera DXM 1200; Stereo Investigator Software, MicroBrightField Europe e.K., Magdeburg, Germany). The optical fractionator method was applied for cell number estimations in the substantia nigra pars compacta (SNc) on the lesioned and non-lesioned side. Volumetric approximations of QA lesion, remaining and contralateral striatal size, as well as ventricles were performed by measuring the surface areas on six consecutives DARPP-32 stained sections on the anterior–posterior axis on the ipsi- and contralateral side. Then the volume was calculated by correcting for section thickness and sample frequency by the following formula: volume (mm^3^) = sum of areas (mm^2^) x 40 μm x 36.

### Data analyses

Statistical analyses were performed using IBM SPSS Statistics 25. Data in text are expressed as mean ± standard deviation. Shapiro Wilk test was used to assess the distribution of data. Data of substance induced rotation, cylinder test, backhand stepping test and histology were distributed normally, while data of forehand stepping test were non-parametric. Accordingly, parametric and non-parametric tests were applied as appropriate.

Overall group differences were evaluated using one-way ANOVA or Kruskal Wallis test with the Bonferroni post-hoc test or Mann Whitney U test with corrections for multiple comparisons.

Contralateral motor deficits were analysed comparing right (ipsilateral to the lesion) versus left (contralateral) motor performance of the forepaws. The motor deficit in cylinder test was evaluated by comparison of the number of wall contacts performed with the contralateral paw and the number of wall contacts performed with the ipsilateral paw at S1 and for stepping test, the motor impairment was assessed by comparison of the number of contralateral and ipsilateral adjustment steps at S1.

For further analyses including QA lesion effect, determination of L-dopa response patterns and correlation analyses the variables were converted into “contralateral paw use” (= contralateral wall contacts divided by the sum of ipsi- plus contralateral wall contacts) for cylinder test and “LAS” (ipsilateral adjustment steps minus contralateral steps divided by the sum of ipsilateral and contralateral steps) for stepping test.

The QA lesion effect (comparing S1 and S2) and L-dopa response (comparing LD1 and LD2) were assessed by using repeated measures ANOVA with post-hoc Bonferroni test and Wilcoxon signed-rank test with correction for multiple comparisons. Within group effects were analysed using t-test for related samples with Bonferroni correction.

Histological data were analysed using one-way ANOVA and student´s t test. Pearson and Spearman correlations were used to specify the relationship between histological parameters and behavioural data. The significance level was set *p*-value <0.05.

## Results

### Amphetamine induced rotation was unaffected by the additional QA lesions

Following 6-OHDA lesion all animals revealed a strong ipsilateral rotation bias with a mean rotation rate of 7.59±3.90 ipsilateral body turns per minute. After randomization into the three experimental groups, mean ipsilateral rotations rates were as follows: 6.93±2.32 in group 1, 6.94±2.67 in group 2, and 7.06±3.04 in group 3 (at this time point only 6-OHDA lesion had been performed and the groups were comparable p = 1). After the QA administration in the groups 1 and 2, amphetamine induced rotation did not show a significant QA lesion effect (F_(1,51)_ = 1.235 p = 0.272).

### A unique rotation behaviour was reflected by apomorphine induced rotation

In group 1 14/19 (74%) animals rotated to the ipsilateral side with a mean rotation rate of 5.62±2.93, 2/19 (11%) rotated at a very low rate (1.54±0.51) towards the contralateral side and 3/19 (16%) did not rotate to any side. In the group 2 11/18 (61%) animals rotated to the ipsilateral side with a mean rotation rate of 6.88±2.74 and 7/18 (39%) rotated to the contralateral side with a mean rotation rate of 4.51±2.43. In contrast, animals of group 3 rotated to the contralateral side exclusively with a mean rotation rate of 4.44±1.52.

### The L-dopa treatment effect was significant at the first behavioural assessment

#### Cylinder test

**Fig 2:**
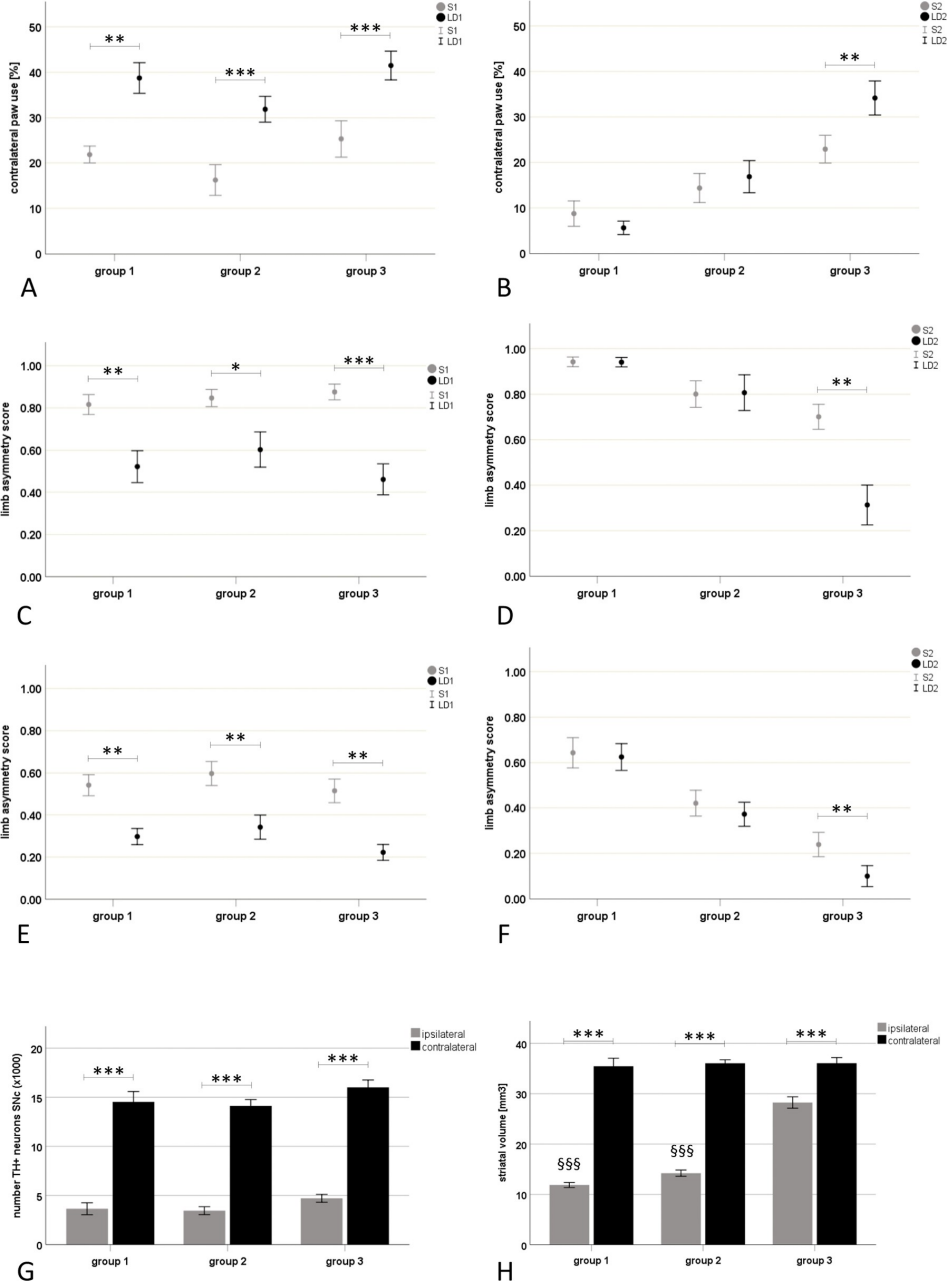
L-dopa response patterns (A-F) and related histology (G-H). Data are illustrated as mean ± standard error. L-dopa responsiveness was evaluated by cylinder and stepping test. All animals received the initial 6-hydroxydopamine (6-OHDA) lesion prior to the first behavioural assessment, while only groups 1 and 2 received the additional quinolinic acid (QA) lesions before the second behavioural assessment: group 1: 6-OHDA+severe QA; group 2: 6-OHDA+mild QA; group 3: 6-OHDA. At the first behavioural assessment (A,C,E), all experimental groups showed a significant motor improvement after L-dopa administration compared to saline treatment in contralateral paw use of cylinder test (A), and limb asymmetry score (LAS) of forehand (C) and backhand stepping test (E; p<0.05). At the second behavioural assessment (B,D,F), the L-dopa treatment effect was still significant in cylinder and stepping test, but declined comparably in both MSA-P/SND groups so that only the group 3 showed a sustained motor improvement during dopaminergic stimulation in both motor tests (p<0.05). G-H: The 6-OHDA lesion resulted in a significant reduction of tyrosine hydroxylase positive (TH+) neurons in the ipsilateral substantia nigra pars compacta (SNc) compared to the contralateral SNc (p<0.001; G). Ipsilateral striatal volume was significantly reduced compared to the contralateral side in all groups (***p<0.001) and the QA lesion resulted in a significant reduction of ipsilateral striatal volumes in groups 1 and 2 compared to the 6-OHDA only lesioned group 3 (^§§§^p<0.001; H). Abbreviations: S1…saline treatment at the first behavioural assessment, LD1…L-dopa treatment at the first behavioural assessment; S2… saline treatment at the second behavioural assessment, LD2…L-dopa treatment at the second behavioural assessment; LAS…limb asymmetry score.

The 6-OHDA lesion resulted in a significant reduction of contralateral paw use as compared to the ipsilateral side (p<0.001; S1 Table) with a mean contralateral paw use of 21.75±11.16% in all lesioned animals without any difference between the experimental groups (S2 Table). L-dopa treatment revealed a significant improvement of contralateral paw use (treatment effect: F_(1,25)_ = 92.884 p<0.001) without an interaction of treatment and group (F_(1,15)_ = 0.041 p = 0.960). Accordingly, the within group analyses confirmed the treatment effect comparing saline and L-dopa behaviour in all experimental groups (group 1 p = 0.003, group 2 p<0.001, group 3 p<0.001; Fig 2A; S2 Table).

#### Stepping test

Following 6-OHDA lesion, there was a significant reduction in adjustment steps performed with the left (contralateral to the 6-OHDA lesion) paw as compared to the right (ipsilateral to the lesion) paw in forehand and backhand directions (p<0.001; S3 and S4 Tables) with a mean LAS of 0.85±0.18 in forehand and 0.55±0.23 in backhand directions (S5 and S6 Tables). At this point, all animals had received only the 6-OHDA lesion and correspondingly, no difference between the experimental groups were detected. Forehand stepping performance was more impaired than backhand stepping (p<0.001). The L-dopa treatment effect was significant in all experimental groups in both forehand and backhand directions (treatment effect in forehand direction: group 1 p = 0.003; group 2 p = 0.021, group 3 p<0.001; backhand direction: F_(1,50)_ = 79.569 p<0.001; group 1 p = 0.003, group 2 p = 0.003, group 3 p = 0.003). No interaction of treatment and group could be identified in backhand direction (F_(2,50)_ = 0.242, p = 0.786) and there were no differences between the experimental groups in either direction (Fig 2C and 2E; S5 and S6 Tables).

### The L-dopa treatment effect declined in both MSA groups at the second behavioural assessment

#### Cylinder test

Comparing contralateral paw use of the first and the second behavioural assessment during saline treatment (S1 versus S2), a significant QA lesion effect was identified (lesion effect F_(1,25)_ = 6.491 p = 0.017), which was attributed to the group 1 without any significant difference to the mild MSA-P/SND or PD/6-OHDA groups. Comparing saline and L-dopa challenge during the 2^nd^ assessment, the L-dopa treatment effect remained significant (treatment effect F_(1,25)_ = 5.122 p = 0.033) and a significant interaction of L-dopa treatment and group was detected (interaction of treatment and group F_(1,25)_ = 7.889 p = 0.002)—both MSA-P/SND groups were different from the group 3 (p<0.05). Within group comparisons assigned the significant L-dopa treatment effect to group 3 (p = 0.003) and no such treatment response could be attributed to group 1 (p = 0.96) or group 2 (p = 1; Fig 2B; S2 Table).

#### Stepping test

Comparing motor performance in stepping test at the first and second behavioural assessment revealed a significant “QA lesion/time” effect wherein a significant improvement was observed in backhand stepping performance (F_(1,51)_ = 8.370 p = 0.006; interaction time and of group: F_(1,51)_ = 7.831 p = 0.001). In this regard, the within group analyses showed that the groups 2 (p = 0.039) and 3 (p = 0.012) improved their backhand stepping performance over the experimental time in contrast to the group 1. At the second behavioural assessment, the L-dopa treatment effect remained significant in group 3 in forehand (p = 0.003) and backhand (p = 0.006) directions (treatment effect in backhand direction: F_(1,51)_ = 6.182 p = 0.016) in contrast to groups 1 and 2 (p = 1). Hence, both MSA-P/SND groups were different from the PD/6-OHDA group (p<0.05) (Fig 2D and 2F; S5 and S6 Tables).

### Number of dopaminergic neurons in SNc was comparable among all groups

The number of tyrosine hydroxylase positive neurons in SNc was significantly reduced ipsilateral to the 6-OHDA lesion compared to the contralateral side (F_(1,48)_ = 603.266; p<0.001) without any significant difference between the experimental groups.

Mean number of dopaminergic neurons accounted for 25% of contralateral side in group 1 (ipsilateral 3649±2509, contralateral 14524±4361), 24% in group 2 (ipsilateral 3450±1710, contralateral 14116±2738) and 31% in group 3 (ipsilateral 4710±1598, contralateral 16005±3003; Fig 2G).

### QA lesion volume was associated with the concentration and coordinates of QA toxin

**Fig 3:**
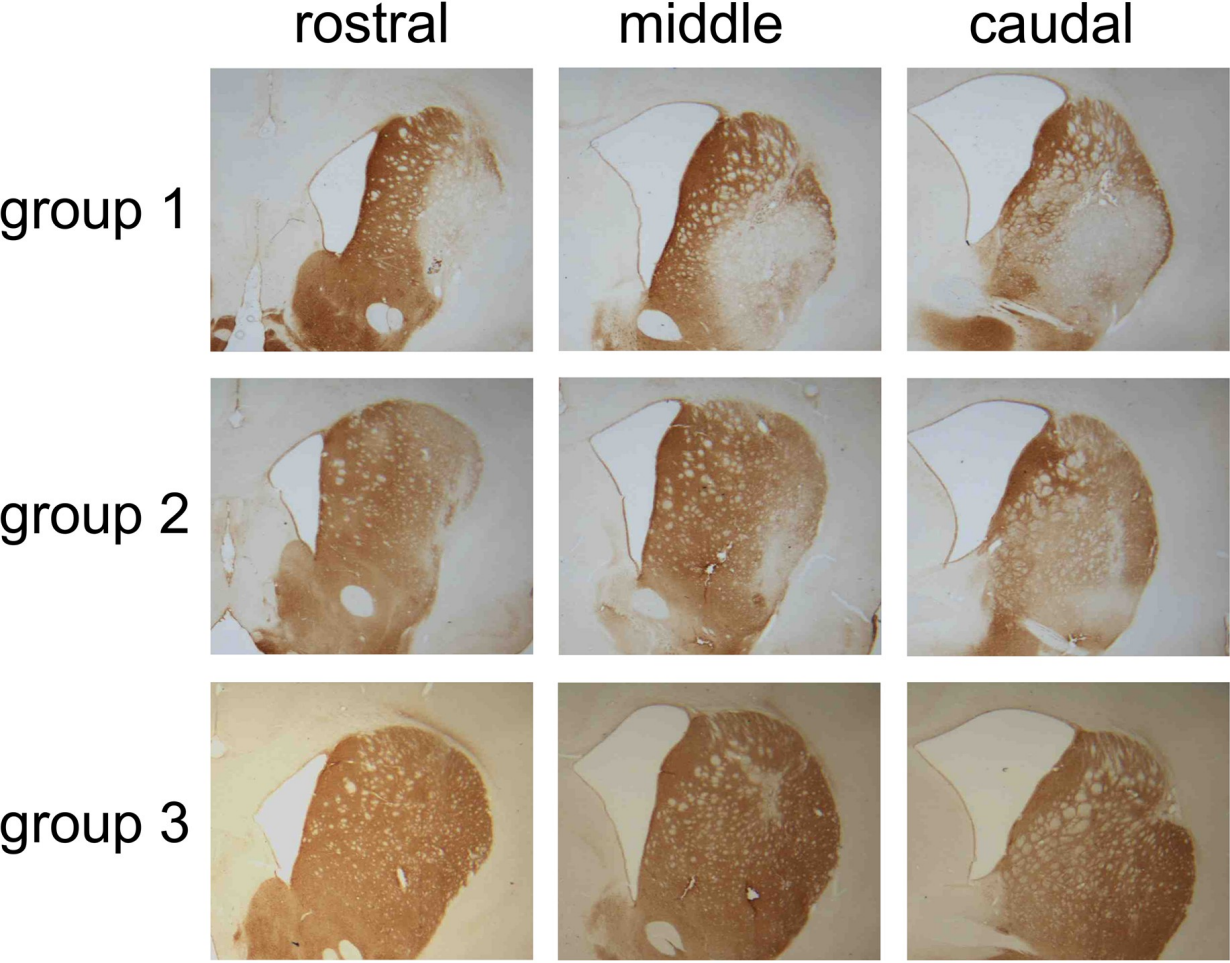
Representative examples of the striatal pathology. Dopamine- and adenosine 3,5-monophosphate regulated phosphoprotein (DARPP32) immunohistochemistry was used to visualize the striatal projection neurons. Three sections in the rostral, middle and caudal section of the striatum are shown per group.

Volumetric measurements on DARPP-32 immunohistochemistry revealed that the QA lesion volume was significantly greater in group 1 as compared to group 2 (t_(33)_ = 5.101 p<0.001). The QA lesion volume was 16.79±3.20 mm^3^ accounting for 46% of contralateral striatal volume in group 1 and 11.28±2.09 mm^3^ accounting for 31% of contralateral striatal volume in group 2. Ipsilateral striatal volume was not only reduced by the mild and severe QA lesions (p<0.001), but also the 6-OHDA lesion lead to significant striatal atrophy as compared to the contralateral side (p<0.001). The remaining striatal volumes of double lesioned animals accounted for 35% of contralateral striatal volume in the group 1 (ipsilateral 12.14±1.80 mm^3^, contralateral 35.45±6.76 mm^3^), and 45% in the group 2 (ipsilateral 14.39±2.54 mm^3^, contralateral 36.05±2.87 mm^3^) compared to 79% in the group 3 (ipsilateral 27.50±4.93 mm^3^, contralateral 36.06±4.60 mm^3^; p<0.001; Fig 2H). Dilatation of the ipsilateral ventricle compared to the contralateral side was present in all lesion groups (F_(1,49)_ = 114.565 p<0.001), without any group effect (group 1: 218.43±11.65%, group 2: 189.68±12.36%; group 3: 177.16±11.65%). Representative examples of the severe and the mild QA lesion of groups 1 and 2 are illustrated in the DARPP32 stain of Fig 3.

### Behavioural and histological correlations

The remaining ipsilateral striatal volume correlated positively with apomorphine induced contralateral rotation behaviour (r = 0.600 p<0.001) and negatively with apomorphine induced ipsilateral rotation behaviour (r = 0.591 p<0.001). The remaining ipsilateral striatal volume also strongly correlated with contralateral paw use during dopaminergic stimulation in cylinder test (LD2; r = 0.798 p<0.001) and forehand (r = 0.788 p<0.001) and backhand stepping (r = .605 p<0.001). Further, apomorphine induced contralateral rotation behaviour correlated with contralateral paw use during L-dopa treatment in cylinder (r = 402 p = 0.017) and stepping test (forehand direction: r = 0.551 p<0.001; backhand direction: r = 0.475 p<0.001).

## Discussion

Previously we have shown that partial dopaminergic denervation and excitotoxic lesions of the lateral motor striatum cause abnormalities of drug-induced circling and spontaneous complex motor deficits in a double lesion rat model of early stage MSA-P/SND [30]. We here demonstrate that L-dopa response failure is similar in the mild and advanced stage MSA-P/SND model. Since the restricted neuropathology achieved by this partial lesion paradigm more closely replicates human MSA-P, and because early treatment intervention is essential in a rapidly progressive neurodegenerative disease like MSA, the mild MSA-P/SND model represents the more appropriate testbed for studying restoration of striatal circuitry using cell grafts.

The intended goal is to restore L-dopa response and thereby improve motor function and quality of life of MSA patients. In the past, several advanced MSA-P/SND models were generated by double-lesion protocols using 6-OHDA and QA resulting in almost complete loss of dopaminergic neurons in the SNc (and ventral tegmental area) and extensive (~40–60%) loss of striatal surface [21–25,37]. To our knowledge, L-dopa responsiveness in forelimb use (stepping and cylinder test) has only been explored in the advanced MSA-P/SND model demonstrating that the extensive neuronal loss of SND results in L-dopa treatment failure and L-dopa induced dyskinesias [19,21]. However, the substantial neurodegeneration of the advanced SND model doesn’t reflect early stage disease and interventional approaches including neuroprotective agents and striatal grafts might be limited.

Studies of L-dopa response patterns in an early MSA-P/SND model are non-existent, but mandatory as testbeds for future neuro-restorative studies. To this end, we here explored two double-lesion approaches modelling different stages of MSA-P/SND in rats as the aim was to create a stable model with restricted striatal and nigral damage combined with initial L-dopa response and secondary L-dopa failure replicating motor and histological characteristics of early MSA-P/SND with PD like onset. Three lesion groups were created including a PD group receiving only the 6-OHDA lesion without any QA lesion (group 3) and two QA lesion approaches differing in QA concentration and striatal coordinates were investigated. The initial 6-OHDA lesion was effective in achieving a contralateral motor deficit of forelimb use in the cylinder and stepping test, whereat the limb asymmetry in stepping test was more impaired in forehand than in backhand direction. The additional QA lesions did not deteriorate stepping performance consistent with previous reports [21,30,37]. However, in cylinder test the more extensive QA lesion resulted in a further reduction of contralateral paw use, albeit no difference to the mild QA lesion or the PD group could be evaluated. The significant L-dopa treatment effect after 6-OHDA lesion declined in both MSA-P/SND groups after the additional mild and more extensive, severe QA lesions. Despite a significant difference in QA lesion volumes between the two MSA groups, no significant differences in the remaining striatal volumes and accordingly, no difference in motor performance comparing saline and L-dopa treatment were observed. However, the remaining striatal volumes correlated strongly with contralateral paw use in cylinder and stepping test during L-dopa challenge. Taken together, the smaller QA lesion (group 2) on top of the 6-OHDA lesion successfully abolished the L-dopa response. Hence, our data strengthen the experimental evidence that the dorsolateral striatum is essential for L-dopa response [21]. Further, our results suggest that the intra-striatal location of the QA lesion and to a lesser extend the QA concentration and thereby the QA lesion volume are decisive in motor performance during dopaminergic stimulation in the double lesion rat model of MSA-P.

A comparison of drug induced rotation by apomorphine prior and post QA lesion surgery was not performed in this study in order to reduce dopamine receptor sensitization [38]. Instead, it was performed at the very end of the study demonstrating a distinctive rotation behaviour of double-lesioned animals reflecting their striatal lesion size: animals of the severe MSA-P/SND-P group demonstrated a strong ipsilateral rotation bias (74% of animals rotated to the ipsilateral side) whereas animals of the MSA-P/SND mild group showed split rotational behaviour (61% rotated ipsilaterally, 39% rotated contralaterally). Contralateral rotation behaviour induced by apomorphine in dopamine depleted animals is thought to be due to the development of dopamine receptor supersensitivity within the denervated striatum of 6-OHDA-lesioned animals [39] and was observed in our 6-OHDA only lesioned animals. It correlated with contralateral motor performance during dopaminergic stimulation in cylinder and stepping tests as well as with the remaining striatal volume. In previous reports apomorphine-induced contralateral rotation behaviour in animals with a unilateral 6-OHDA lesion of the MFB was abolished by an additional striatal QA lesion [23,24, 40–42]. In line with previous work performing partial striatal or MFB 6-OHDA lesions, amphetamine induced ipsiversive rotations persisted after the subsequent QA lesions. Further, amphetamine induced rotations correlated with the number of TH positive cells in the SNc and failed to distinguish between single (only 6-OHDA) and double (6-OHDA plus QA) lesioned animals [21,22,25,30,37,40,43]. Ipsilateral rotation has been observed in QA treated animals replicating striatal lesions of Huntington`s disease [30,44,45].

Histologically the MSA-P/SND groups were easily distinguished according to their different QA lesion volumes. However, no differences in remaining striatal volume or ventricular dilatation were observed between the two MSA groups which is reflected by the comparable behavioural results (discussed above). Intrastriatal 6-OHDA lesion resulted in ~75% loss of dopaminergic cells in the SNc in all lesion groups in contrast to ~90% loss in our previous work [22] reflecting the variability of striatal 6-OHDA lesions. The subsequent QA lesions did not affect the number of dopaminergic cells in SNc consistent with previous reports [19].

### Limitations

No QA sham lesion was performed in group 3 on top of the 6-OHDA lesion. Accordingly, the striatal damage induced by the 6-OHDA lesion in the PD group was not controlled and the L-dopa effect on motor function may have been attenuated in these animals.

## Conclusion

We here characterize for the first time L-dopa response patterns in a partial double-lesion rat model of early stage MSA-P/SND with limited dopaminergic nigrostriatal denervation and striatal volume loss. Our mild MSA-P/SND double-lesion model provides a highly suitable testbed for cell-based restoration of striatal circuitry and reversal of L-dopa response failure.

## Supporting information

S1 Table **Number of wall contacts performed with the left (contralateral) and the right (ipsilateral to the lesions) paw in cylinder test.** Data are presented as means ± standard deviation; group 1: 6-OHDA+severe QA; group 2: 6-OHDA+mild QA; group 3: 6-OHDA; ***…significantly different from ipsilateral side p<0.001; abbreviations: MSA-P…multiple system atrophy Parkinson variant; SND…striatonigral degeneration; PD…Parkinson´s disease; S1…saline treatment at the first behavioural assessment, LD1…L-dopa treatment at the first behavioural assessment; S2…saline treatment at the second behavioural assessment; LD2…L-dopa treatment at the second behavioural assessment; L…left; R…right.
(DOCX)

S2 Table Contralateral paw use in cylinder test. Data are presented as means ± standard deviation.; group 1: 6-OHDA+severe QA; group 2: 6-OHDA+mild QA; group 3: 6-OHDA; at the first behavioural assessment, all groups revealed a significant L-dopa treatment effect comparing saline (S1) and L-dopa treatment (LD1) (p<0.01). At the second behavioural assessment, a significant L-dopa treatment effect was attributed to the group 3 (p = 0.003) in contrast to groups 1 and 2; * indicate the level of significance comparing L-dopa versus saline treatment at the first or second behavioural assessment—*** …p<0.001; ** …p<0.01.
(DOCX)

S3 Table **Adjustment steps performed with the right (ipsilateral) and the left (contralateral to the lesion) paw during stepping test in backhand direction.** Data are presented as mean steps ± standard deviation in backhand direction; group 1: 6-OHDA+severe QA; group 2: 6-OHDA+mild QA; group 3: 6-OHDA; ***…significantly different from ipsilateral side p<0.001. Abbreviations: MSA-P…multiple system atrophy Parkinson variant; SND…striatonigral degeneration; PD…Parkinson´s disease; S1…saline treatment at the first behavioural assessment, LD1…L-dopa treatment at the first behavioural assessment; S2…saline treatment at the second behavioural assessment; LD2…L-dopa treatment at the second behavioural assessment; L…left; R…right.
(DOCX)

S4 Table **Adjustment steps performed with the left (contraateral) and the right (ipsilateral to the lesion) paw during stepping test in forehand direction.** Data are presented as mean steps ± standard deviation in forehand direction; group 1: 6-OHDA+severe QA; group 2: 6-OHDA+mild QA; group 3: 6-OHDA; ***…significantly different from ipsilateral side p<0.001. Abbreviations: MSA-P…multiple system atrophy Parkinson variant; SND…striatonigral degeneration; PD…Parkinson´s disease; S1…saline treatment at the first behavioural assessment, LD1…L-dopa treatment at the first behavioural assessment; S2…saline treatment at the second behavioural assessment; LD2…L-dopa treatment at the second behavioural assessment; L…left; R…right.
(DOCX)

S5 Table Limb asymmetry in stepping test in backhand direction. Data are presented as mean limb asymmetry score (LAS) ± standard deviation; group 1: 6-OHDA+severe QA; group 2: 6-OHDA+mild QA; group 3: 6-OHDA. At the first behavioural assessment, all 6-OHDA lesioned animals revealed a significant L-dopa treatment effect comparing saline (S1) and L-dopa treatment (LD1) (p<0.001). At the second behavioural assessment, a significant L-dopa treatment effect was attributed to the Group 3 (p = 0.012) in contrast to MSA-P/SND groups; * indicate the level of significance comparing L-dopa versus saline treatment at the first and the second behavioural assessment—*** …p<0.001; ** …p<0.01; * …p<0.05. Abbreviations: MSA-P…multiple system atrophy Parkinson variant; SND…striatonigral degeneration; PD…Parkinson´s disease; S1…saline treatment at the first behavioural assessment, LD1…L-dopa treatment at the first behavioural assessment; S2…saline treatment at the second behavioural assessment; LD2…L-dopa treatment at the second behavioural assessment.
(DOCX)

S6 Table Limb asymmetry score in stepping test in forehand direction. Limb asymmetry score during saline (S1 and S2) and L-Dopa challenge (LD1 and LD2) in stepping test forehand direction. Data are presented as mean limb asymmetry score (LAS) ± standard deviation; group 1: 6-OHDA+severe QA; group 2: 6-OHDA+mild QA; group 3: 6-OHDA. At the first behavioural assessment, all groups revealed a significant L-dopa treatment effect comparing saline (S1) and L-dopa treatment (LD1) (p<0.001). At the second behavioural assessment, a significant L-dopa treatment effect was attributed to the group 3 (p = 0.003) in contrast to MSA-P/SND groups; * indicate the level of significance comparing L-dopa versus saline treatment at the first or second behavioural assessment—*** …p<0.001; ** …p<0.01. Abbreviations: MSA-P…multiple system atrophy Parkinson variant; SND…striatonigral degeneration; PD…Parkinson´s disease; S1…saline treatment at the first behavioural assessment, LD1…L-dopa treatment at the first behavioural assessment; S2…saline treatment at the second behavioural assessment; LD2…L-dopa treatment at the second behavioural assessment.
(DOCX)
