# Supplementary material for: L-dopa response pattern in a rat model of mild striatonigral degeneration
Source: PLoS One. 2019 Jun 10;14(6):e0218130. doi: 10.1371/journal.pone.0218130 (PMC6557500; doi:10.1371/journal.pone.0218130)
Supplement: S1 Table — Number of wall contacts performed with the left (contralateral) and the right (ipsilateral to the lesions) paw in cylinder test. Data are presented as means ± standard deviation; group 1: 6-OHDA+severe QA; group 2: 6-OHDA+mild QA; group 3: 6-OHDA; ***…significantly different from ipsilateral side p<0.001; abbreviations: MSA-P…multiple system atrophy Parkinson variant; SND…striatonigral degeneration; PD…Parkinson´s disease; S1…saline treatment at the first behavioural assessment, LD1…L-dopa treatment at the first behavioural assessment; S2…saline treatment at the second behavioural assessment; LD2…L-dopa treatment at the second behavioural assessment; L…left; R…right. (DOCX) [file pone.0218130.s001.docx]

|  | **S1** | | **LD1** | | **S2** | | **LD2** | |
| --- | --- | --- | --- | --- | --- | --- | --- | --- |
|  | **L** | **R** | **L** | **R** | **L** | **R** | **L** | **R** |
| Group 1 | 4.38±.06*** | 15.63±1.06 | 7.60±1.82 | 12.40±1.82 | 2.20±3.90 | 17.80±3.90 | 1.00±1.00 | 19.00±1.00 |
| Group 2 | 3.25±1.91*** | 16.75±1.91 | 5.17±2.48 | 14.83±2.48 | 2.33±1.51 | 17.66±1.51 | 3.17±2.14 | 16.83±2.00 |
| Group 3 | 4.92±2.61*** | 14.75±3.11 | 6.60±1.82 | 13.00±2.71 | 4.30±2.50 | 15.70±2.50 | 7.00±2.58 | 13.00±2.58 |
