# Supplementary material for: L-dopa response pattern in a rat model of mild striatonigral degeneration
Source: PLoS One. 2019 Jun 10;14(6):e0218130. doi: 10.1371/journal.pone.0218130 (PMC6557500; doi:10.1371/journal.pone.0218130)
Supplement: S2 Table — Data are presented as means ± standard deviation.; group 1: 6-OHDA+severe QA; group 2: 6-OHDA+mild QA; group 3: 6-OHDA; at the first behavioural assessment, all groups revealed a significant L-dopa treatment effect comparing saline (S1) and L-dopa treatment (LD1) (p<0.01). At the second behavioural assessment, a significant L-dopa treatment effect was attributed to the group 3 (p = 0.003) in contrast to groups 1 and 2; * indicate the level of significance comparing L-dopa versus saline treatment at the first or second behavioural assessment—*** …p<0.001; ** …p<0.01. (DOCX) [file pone.0218130.s002.docx]

|  | **S1** | | LD1 | | S2 | LD2 |
| --- | --- | --- | --- | --- | --- | --- |
| Group 1 | 21.88±5.30 | 21.75±11.16 | 38.75±9.54** | 33.68±2.25*** | 8.75±7.91 | 5.63±4.17 |
| Group 2 | 16.25±9.54 |  | 31.88±7.99*** |  | 14.38±9.04 | 16.88±9.98 |
| Group 3 | 25.33±13.93 |  | 41.50±10.98*** |  | 22.92±10.54 | 34.17±12.94** |
