# Supplementary material for: L-dopa response pattern in a rat model of mild striatonigral degeneration
Source: PLoS One. 2019 Jun 10;14(6):e0218130. doi: 10.1371/journal.pone.0218130 (PMC6557500; doi:10.1371/journal.pone.0218130)
Supplement: S3 Table — Adjustment steps performed with the right (ipsilateral) and the left (contralateral to the lesion) paw during stepping test in backhand direction. Data are presented as mean steps ± standard deviation in backhand direction; group 1: 6-OHDA+severe QA; group 2: 6-OHDA+mild QA; group 3: 6-OHDA; ***…significantly different from ipsilateral side p<0.001. Abbreviations: MSA-P…multiple system atrophy Parkinson variant; SND…striatonigral degeneration; PD…Parkinson´s disease; S1…saline treatment at the first behavioural assessment, LD1…L-dopa treatment at the first behavioural assessment; S2…saline treatment at the second behavioural assessment; LD2…L-dopa treatment at the second behavioural assessment; L…left; R…right. (DOCX) [file pone.0218130.s003.docx]

|  | **S1** | | **LD1** | | **S2** | | **LD2** | |
| --- | --- | --- | --- | --- | --- | --- | --- | --- |
|  | **L** | **R** | **L** | **R** | **L** | **R** | **L** | **R** |
| Group 1 | 3.10±1.78*** | 9.66±1.16 | 5.26±1.78 | 9.38±0.78 | 2.37±2.17 | 8.97±1.15 | 2.44±2.07 | 8.91±1.21 |
| Group 2 | 2.78±2.12*** | 9.87±1.27 | 5.16±2.40 | 9.97±2.83 | 3.93±2.24 | 8.94±0.93 | 3.95±1.58 | 8.86±1.07 |
| Group 3 | 3.35±2.23*** | 9.31±1.28 | 6.06±1.92 | 9.24±1.46 | 5.60±2.29 | 8.68±1.13 | 7.11±1.99 | 8.47±1.41 |
