# Supplementary material for: L-dopa response pattern in a rat model of mild striatonigral degeneration
Source: PLoS One. 2019 Jun 10;14(6):e0218130. doi: 10.1371/journal.pone.0218130 (PMC6557500; doi:10.1371/journal.pone.0218130)
Supplement: S4 Table — Adjustment steps performed with the left (contraateral) and the right (ipsilateral to the lesion) paw during stepping test in forehand direction. Data are presented as mean steps ± standard deviation in forehand direction; group 1: 6-OHDA+severe QA; group 2: 6-OHDA+mild QA; group 3: 6-OHDA; ***…significantly different from ipsilateral side p<0.001. Abbreviations: MSA-P…multiple system atrophy Parkinson variant; SND…striatonigral degeneration; PD…Parkinson´s disease; S1…saline treatment at the first behavioural assessment, LD1…L-dopa treatment at the first behavioural assessment; S2…saline treatment at the second behavioural assessment; LD2…L-dopa treatment at the second behavioural assessment; L…left; R…right. (DOCX) [file pone.0218130.s004.docx]

|  | **S1** | | **LD1** | | **S2** | | **LD2** | |
| --- | --- | --- | --- | --- | --- | --- | --- | --- |
|  | **L** | **R** | **L** | **R** | **L** | **R** | **L** | **R** |
| Group 1 | 0.86±1.04*** | 7.54±1.41 | 2.89±2.71 | 7.54±1.28 | 0.25±0.41 | 7.04±1.76 | 0.24±0.36 | 7.00±1.88 |
| Group 2 | 0.74±0.88*** | 7.97±1.67 | 2.12±2.13 | 7.92±3.15 | 1.00±1.95 | 6.95±2.06 | 0.85±1.72 | 6.68±2.49 |
| Group 3 | 0.54±0.75*** | 6.92±2.17 | 1.81±2.05 | 7.10±1.29 | 1.56±2.23 | 7.02±1.88 | 3.47±2.82 | 6.12±2.53 |
