# Supplementary material for: L-dopa response pattern in a rat model of mild striatonigral degeneration
Source: PLoS One. 2019 Jun 10;14(6):e0218130. doi: 10.1371/journal.pone.0218130 (PMC6557500; doi:10.1371/journal.pone.0218130)
Supplement: S5 Table — Data are presented as mean limb asymmetry score (LAS) ± standard deviation; group 1: 6-OHDA+severe QA; group 2: 6-OHDA+mild QA; group 3: 6-OHDA. At the first behavioural assessment, all 6-OHDA lesioned animals revealed a significant L-dopa treatment effect comparing saline (S1) and L-dopa treatment (LD1) (p<0.001). At the second behavioural assessment, a significant L-dopa treatment effect was attributed to the Group 3 (p = 0.012) in contrast to MSA-P/SND groups; * indicate the level of significance comparing L-dopa versus saline treatment at the first and the second behavioural assessment—*** …p<0.001; ** …p<0.01; * …p<0.05. Abbreviations: MSA-P…multiple system atrophy Parkinson variant; SND…striatonigral degeneration; PD…Parkinson´s disease; S1…saline treatment at the first behavioural assessment, LD1…L-dopa treatment at the first behavioural assessment; S2…saline treatment at the second behavioural assessment; LD2…L-dopa treatment at the second behavioural assessment. (DOCX) [file pone.0218130.s005.docx]

|  | **S1** | | **LD1** | | **S2** | **LD2** |
| --- | --- | --- | --- | --- | --- | --- |
| Group 1 | 0.54±0.22 | 0.55±0.23 | 0.30±0.17** | 0.29±0.19*** | 0.64±0.29 | 0.62±0.26 |
| Group 2 | 0.60±0.24 |  | 0.34±0.24** |  | 0.41±0.25 | 0.39±0.21 |
| Group 3 | 0.51±0.23 |  | 0.22±0.16** |  | 0.24±0.22 | 0.10±0.19* |
